# The Potential of Current- and Wind-Driven Transport for Environmental Management of the Baltic Sea

**DOI:** 10.1007/s13280-013-0486-3

**Published:** 2014-01-12

**Authors:** Tarmo Soomere, Kristofer Döös, Andreas Lehmann, H. E. Markus Meier, Jens Murawski, Kai Myrberg, Emil Stanev

**Affiliations:** 9 Institute of Cybernetics at Tallinn University of Technology, Akadeemia tee 21, 12618 Tallinn, Estonia; 1 Estonian Academy of Sciences, Kohtu 6, 10130 Tallinn, Estonia; 2 Department of Meteorology, Stockholm University, 106 91 Stockholm, Sweden; 3 GEOMAR Helmholtz Centre for Ocean Research Kiel, Düsternbrooker Weg 20, 24105 Kiel, Germany; 4 Swedish Meteorological and Hydrological Institute, 60176 Norrköping, Sweden; 5 Denmark’s Meteorological Institute (DMI), Lyngbyvej 100, 2100 Copenhagen, Denmark; 6 Finnish Environment Institute/Marine Research Centre, P.O. Box 140, 00251 Helsinki, Finland; 7 Department of Geophysics, Klaipeda University, Herkaus Manto g. 84, Klaipeda, Lithuania; 8 Institute of Coastal Research, Helmholtz Zentrum Geesthacht, Max-Planck-Strasse 1, 21502 Geesthacht, Germany

**Keywords:** Pollution management, Oil pollution, Fairway design, Lagrangian transport, Ocean modeling, Baltic Sea, Gulf of Finland

## Abstract

The ever increasing impact of the marine industry and transport on vulnerable sea areas puts the marine environment under exceptional pressure and calls for inspired methods for mitigating the impact of the related risks. We describe a method for preventive reduction of remote environmental risks caused by the shipping and maritime industry that are transported by surface currents and wind impact to the coasts. This method is based on characterizing systematically the damaging potential of the offshore areas in terms of potential transport to vulnerable regions of an oil spill or other pollution that has occurred in a particular area. The resulting maps of probabilities of pollution to be transported to the nearshore and the time it takes for the pollution to reach the nearshore are used to design environmentally optimized fairways for the Gulf of Finland, Baltic Proper, and south-western Baltic Sea.

## Introduction

Traditionally risks related to maritime industry are associated with potential accidents (ship collisions, sinking or grounding, leaks from oil platforms, etc.) that may lead to loss of lives or property, or to environmental pollution. The management of the related environmental risks has been mostly focused on small areas around the installation or the ship in question. An intrinsic feature of the ocean environment is that various meteorological and oceanic factors (such as currents, winds or waves, called met-ocean drivers below) can transport various impacts over long distances. For example, exhaust emissions (Hobbs et al. 2000), external noise (Slabbekoorn et al. 2010; Merchant et al. 2012), litter and debris (Pichel et al. 2007), and especially oil or chemical pollution may cause large-scale consequences. Large accidents provide substantial risks to the ecosystem even in seemingly remote and safe locations as demonstrated, for example, by the recent Gulf of Mexico oil spill (Camilli et al. 2010) or Tohoku tsunami (Bagulayan et al. 2012).

This component of environmental risk is exceptionally important in small seas that host intense ship traffic such as the Baltic Sea. At present this sea accounts for up to 15 % of the world’s international ship cargo transportation. Sustainable management of this traffic flow is a major challenge there. The largest threat to this region is oil transportation that has increased by more than a factor of two from year 2000 to 2006 (Knudsen 2010).

The traditionally used approach to manage potential maritime pollution is to develop proper decision support systems and quick remedial action plans for the event of an accident (e.g., Keramitsoglou et al. 2003; Kostianoy et al. 2008). Another approach is the preventive planning strategy; for instance, the optimization of the shipping routes (Schwehr and McGillivary 2007), dynamical relocation of tugboats (Eide et al. 2007), or designation of possible policies and regulations (Ko and Chang 2010; Hassler 2011; Rusli 2012). Their aim is to account for the effect that a pollution accident would incur before it actually happens.

A commonly accepted paradigm is that some sea areas (e.g., spawning areas) are more valuable than others (Kachel 2008). In this framework, the cost of environmental consequences of an accident depends on not only on its severity but also on when and at which point the adverse impacts have been released. Therefore, tagging sea areas with price labels naturally yields an associated distribution of costs of otherwise similar accidents but occurring at different locations.

Our goal was to create a technology that would be able to specify such distributions (especially those created by intrinsic favorable features of the marine dynamics) in a particular case when the adverse impact is transported by various met-ocean drivers over the sea surface to predefined vulnerable areas. The associated environmental damage can be minimized preventively by placing maritime activities (like ship routes) in the safest areas (called areas of reduced risk) indicated by these distributions.

A common example of adverse impact is oil pollution and the natural vulnerable area is the coastal zone (Kachel 2008). Alternatively, marine protected areas may be considered as valuable regions (Delpeche-Ellmann and Soomere 2013). A simple measure of the cost of an accident at a particular sea point is the probability with which some vulnerable area is hit by adverse impacts released at this point (Andrejev et al. 2011). Another convenient measure of this cost is the time it takes for this impact to reach a vulnerable area. This quantity, called particle age (Andrejev et al. 2011) or residence time (of oil) at sea (Murawski and Woge Nielsen 2013), is decisive in estimates of the capacity of oil combating services.

The use of favorable features of ocean dynamics for the mitigation of consequences of potential accidents is straightforward in regions hosting persistent currents or steady winds where it is sufficient to place the potentially dangerous activities as far as possible “upstream” or “upwind” from the vulnerable areas (Soomere and Quak 2007). Doing so is not possible in the Baltic Sea that is famous for the transient nature and high variability of the patterns of driving forces, extreme complexity of the dynamics of the marine environment, very small internal Rossby radius (Fennel et al. 1991; Alenius et al. 2003), chaotic appearance and low directional persistence of surface currents (Andrejev et al. 2004a, b), and extremely complicated paths of drifters (Döös and Engqvist 2007).

There exist, however, highly ordered patterns of currents in this sea such as frequently repeating pathways of single particles (Döös et al. 2004) and water masses (Meier 2007), highly persistent flows in certain layers (Andrejev et al. 2004a, b), or semi-persistent patterns of Lagrangian transport (Soomere et al. 2011d). It is likely that such patterns render the probability of transport of various substances or items from different sea areas to vulnerable regions highly variable. The areas, for which this probability is relatively small, or the propagation from which to vulnerable areas takes a long time, are the best candidates for potentially dangerous activities such as ship traffic or offshore structures.

In order to reach the above goal, first of all computer simulations have to provide an adequate statistics of the water motions. The cumulative values of the above-discussed measures (probability or particle age) vary substantially within the first seasons of calculations. They reach an almost constant level after about 4 years in the Gulf of Finland (Andrejev et al. 2011) whereas the pattern of hits to the coasts is exactly the same for the 1980s and 1990s (Viikmäe et al. 2013). Therefore, it is necessary to cover, at least, 5 years in order to reach an acceptable estimate of the “climatological” values (understood here as averages over longer time intervals) of these measures.

Another crucial problem is how to extract useful information from the results of long-term simulations. Moreover, the quantification of the potential of different areas to serve as a source of danger to the vulnerable regions involves solving an inverse problem of pollution propagation. Such problems are frequently mathematically ill-posed and no universal method exists for solving them. This requires the use of non-traditional mathematical methods to identify the effect of favorable patterns on the pollution propagation. An approximate solution to such problems can be obtained by means of statistical analysis of a large number of particular solutions (Lagrangian trajectories) of the direct problem of propagation of pollution parcels (Soomere et al. 2010; Andrejev et al. 2011). Following this logic, the developed method contains four components: (i) a high-resolution circulation model, (ii) a scheme for tracking of Lagrangian trajectories of pollution parcels, (iii) a technique for the calculation of quantities characterizing the potential of different sea areas to supply adverse impacts, and (iv) decision-making routines (Andrejev et al. 2011).

## Modeling Environment

**Fig. 1:**
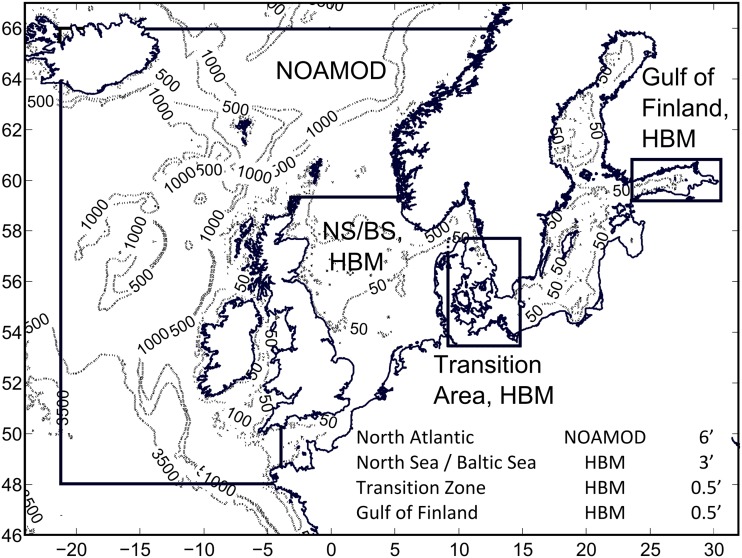
Scheme of three-level nested domains for the modeling of oil drift and fate in the Gulf of Finland using NOAMOD and HBM models (Murawski and Woge Nielsen 2013). An analogous scheme, with no nesting for the Gulf of Finland, was used in Lu et al. (2012). The RCO and BSIOM models were applied to the entire interior of the Baltic Sea. The OAAS model was only applied to the Gulf of Finland. ©Springer International Publishing. Reprinted with kind permission of Springer Science + Business Media

Four different circulation models were used to simulate currents in the Baltic Sea and its sub-basins. The DMI/BSH cmod (Kleine 1994; Dick et al. 2001) was applied to the southern Baltic Sea (Lu et al. 2012). Its further development, the HIROMB-BOOS community (HBM) model, was used to calculate oil drift and fate in the Gulf of Finland (Murawski and Woge Nielsen 2013). A barotropic two-dimensional (2D) surge model NOAMOD with a horizontal resolution of 6 nautical miles and covering a large part of the north-eastern Atlantic provided boundary conditions for a 3D DMI/BSH cmod or HBM North Sea–Baltic Sea model, with a horizontal resolution of 3 nautical miles (about 5.5 km; Fig. 1). A finer model of the transition area between these seas (incl. the south-western Baltic Sea) with a horizontal resolution of 0.5 nautical miles was two-way nested into the above 3-mile model (Lu et al. 2012; Murawski and Woge Nielsen 2013). Another finer 0.5-mile model was applied for the Gulf of Finland (Murawski and Woge Nielsen 2013). These models were forced using the Denmark’s Climate Centre reanalysis (Christensen et al. 2006) for the North Atlantic, North Sea, and Baltic Sea with a spatial and temporal resolution of 0.11° and 1 h, respectively.

Velocity fields calculated using the Rossby Centre Ocean circulation model [RCO, Swedish Meteorological and Hydrological Institute (SMHI)] for the Baltic Sea (Meier 2001, 2007; Meier et al. 2003) were used for the evaluation of the drift of Eulerian tracers in the entire Baltic Sea and for the calculation of Lagrangian trajectories in the northern Baltic Proper (Viikmäe et al. 2011) and in the Gulf of Finland (Soomere et al. 2010, 2011a, b). The horizontal resolution of this model (about 2 nautical miles) is usually sufficient for eddy-resolving runs in the Baltic Proper (Lehmann 1995) but is barely eddy-permitting in the Gulf of Finland (Alenius et al. 2003).

The OAAS model (Andrejev et al. 2004a, b, 2010) was used to simulate the currents in the Gulf of Finland in three horizontal resolutions (2, 1, and 0.5 nautical miles) with otherwise identical setup and vertical resolution. The boundary information at the entrance to the gulf was extracted from the output of the RCO model. The RCO and OAAS models were forced with identical meteorological data from a regionalisation of the ERA-40 reanalysis over Europe during 1961–2007 (Samuelsson et al. 2011).

Alternatively, currents for the entire Baltic Sea, including Skagerrak and the Kattegat, were reconstructed using the Kiel Baltic Sea Ice Ocean model (BSIOM, Lehmann et al. 2002, 2012; Hinrichsen et al. 2009) with a horizontal resolution of 2.5 km. This model was forced by atmospheric conditions from the SMHI meteorological database (Lars Meuller, pers. comm.) which covers the whole Baltic basin on a regular grid of 1° × 1° with a temporal increment of 3 h.

## Trajectory Simulations and Drifter Experiments

The test elements to evaluate the risk of a hit by pollution released at a particular sea point were numerically simulated Lagrangian trajectories of water or pollution parcels. For most of the research presented below their trajectories were not truly Lagrangian: they were locked in the uppermost layer and exerted only horizontal current-driven advection. This approach evidently does not replicate the fate of oil spills that are also affected by other met-ocean drivers, chemical processes, buoyancy effects, Stokes drift, etc. (Fingas 2011; Murawski and Woge Nielsen 2013). It is applicable for persistent substances dissolved in the thin uppermost layer (e.g., different contaminants or radioactive materials) in strongly stratified environments. The benefit from the quasi-Lagrangian models of this type is the possibility to evaluate the contribution of current-driven propagation into the environmental risks. The drift and fate of oil spills under the joint impact of currents and wind were addressed using an advanced oil spill model in the Gulf of Finland (Murawski and Woge Nielsen 2013).

Different models used various ways to construct the Lagrangian trajectories of water and pollution parcels. “Off-line” simulation (in which the circulation modeling was separated from the trajectory calculation) was used to build the trajectories from the output of the RCO model for the Gulf of Finland (Soomere et al. 2011c, d) and the northern Baltic Proper (Viikmäe et al. 2011), from the data of the DMI/BSH cmod model for the south-western Baltic Sea (Lu et al. 2012) and in the research performed using the BSIOM model (Lehmann et al. 2014). In the first three occasions, the trajectories were calculated with the non-spreading version of the TRACMASS code (Döös 1995; Blanke and Raynaud 1997; de Vries and Döös 2001). It semi-analytically reconstructs the motion of the particles in a fully invertible manner but ignores so-called subgrid-scale turbulence. The drawback of the non-spreading scheme is that the initially close-modeled trajectories have a tendency to stay much closer than real drifters (Jönsson et al. 2004; Engqvist et al. 2006; Döös and Engqvist 2007; Döös et al. 2011; Kjellsson and Döös 2012). An implicit consequence is that the trajectories cannot reach the coast and the nearshore had to be defined as a 3 grid cells wide zone near the coast (Viikmäe et al. 2010; Lu et al. 2012). Inclusion of subgrid-scale processes, e.g., by means of adding stochastic elements to single trajectories (Andrejev et al. 2010; Döös et al. 2013), may lead to more adequate spreading of trajectories. However, the added distortions do not mirror the real fluid flow and therefore may substantially impact the appearance of a single trajectory.

The calculations with Eulerian tracers (Höglund and Meier 2012) and simulations with the OAAS model (Andrejev et al. 2010, 2011) used various “on-line” ways of the evaluation of the motion of selected parcels. They were carried out simultaneously with the integration of the circulation model and replicated the impact of subgrid turbulence.

**Fig. 2:**
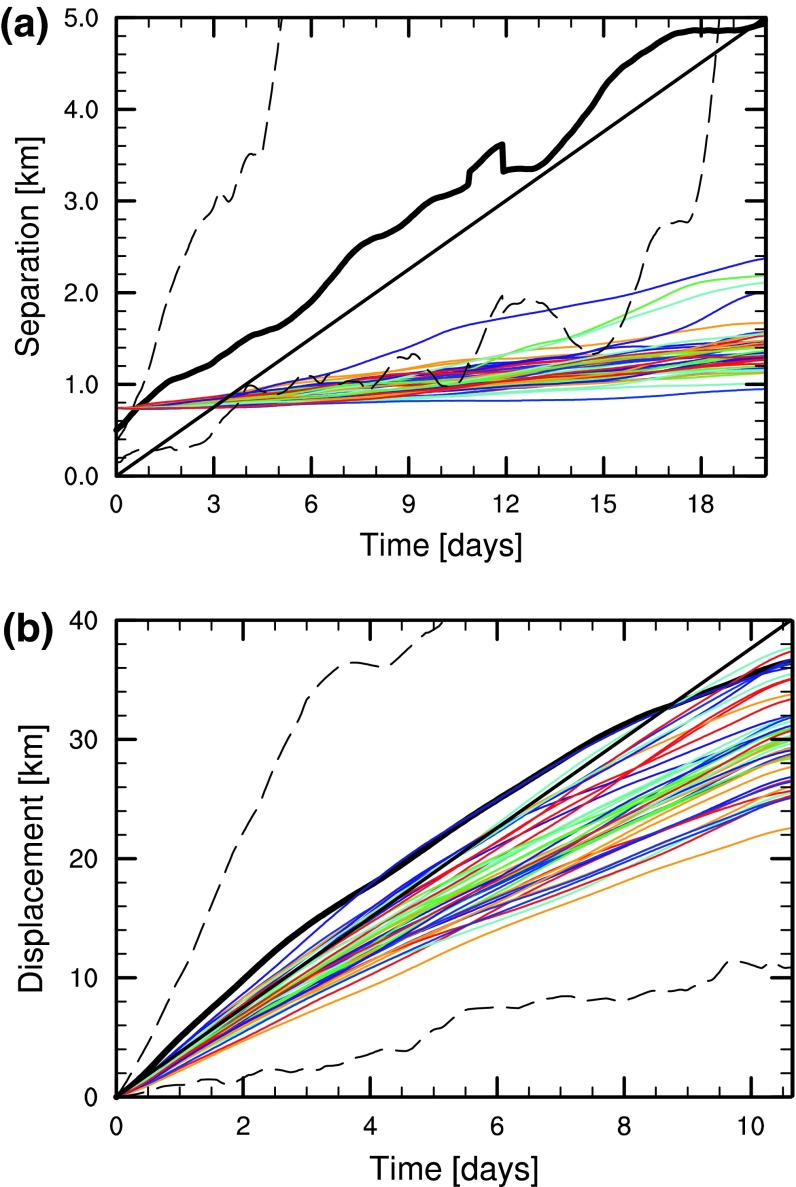
Mean pair separation (**a**) and displacement (**b**) of drifters. *Solid black lines* all segments of real drifters in 2010–2011; *solid colored lines* single segments simulated using the RCO model for each year 1962–2004; *dashed black lines* the 10th and 90th percentiles for segments of trajectories of real drifters (Kjellsson and Döös 2012)

The ability of simulations to reproduce the marine dynamics has been verified using in situ experiments (Soomere et al. 2011e; Kjellsson and Döös 2012). The adequacy of the basic statistical properties of simulated Lagrangian trajectories was estimated using twelve World Ocean Circulation Experiment (WOCE) style subsurface (following currents at depths of 12–18 m) drifters (Kjellsson and Döös 2012). The relative and absolute dispersion as well as the mean displacement of simulated drifters were all significantly lower than those of the real drifters (Fig. 2). Most likely, too weak model currents and the too coarse model grid together reduced the spatial variability of the motions.

This shortage can be partially removed by adding subgrid turbulence into the trajectory calculation (Kjellsson and Döös 2012). A comparison of the patterns of coastal hits in the Gulf of Finland based on the results of the RCO model and several spreading mechanisms revealed that although single trajectories were at times radically modified, the most frequent locations of coastal hits were insensitive with respect to particular spreading mechanisms (Viikmäe et al. 2013). The presence of spreading increased the number of coastal hits by about 1–2 % in single years and reduced the average time it takes for particles to reach the coast by less than 3 %. This feature indicates that the RCO model still adequately represents the most influential patterns of current-driven advection in this area even if several statistical properties of flow velocities are not exactly replicated.

## Areas of Reduced Risk and Fairway Design

**Fig. 3:**
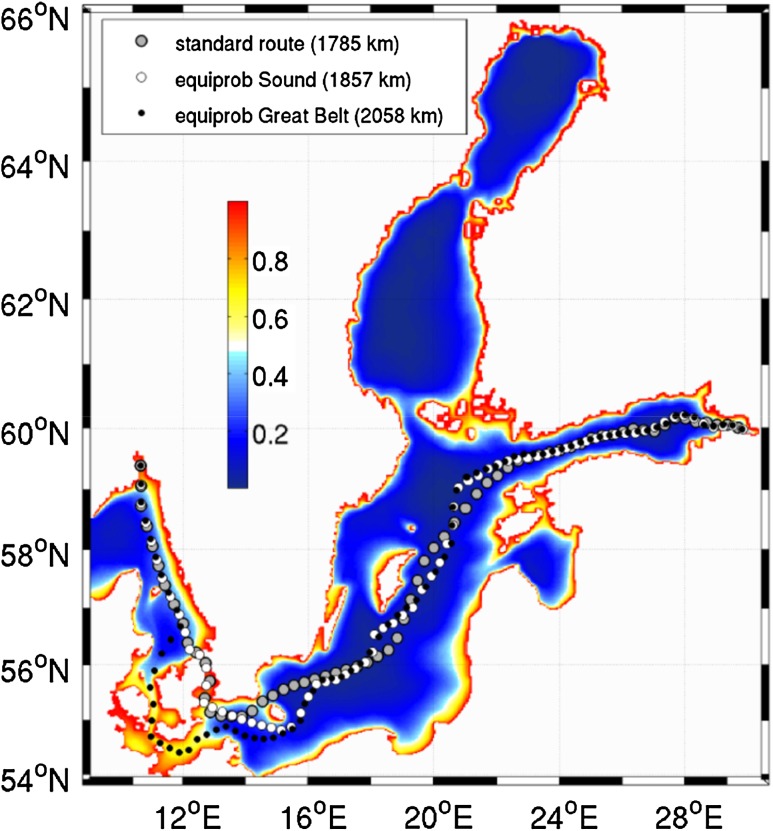
Average probability to reach the coast within 10 days from the instant of release calculated using the BSIOM model for 2002–2010. The *gray dots* indicate the frequently used shipping route from Oslo to Saint Petersburg. Environmentally safer fairways through the Sound and Great Belts are indicated with *white* and *black dots*, respectively (Lehmann et al. 2014)

**Fig. 4:**
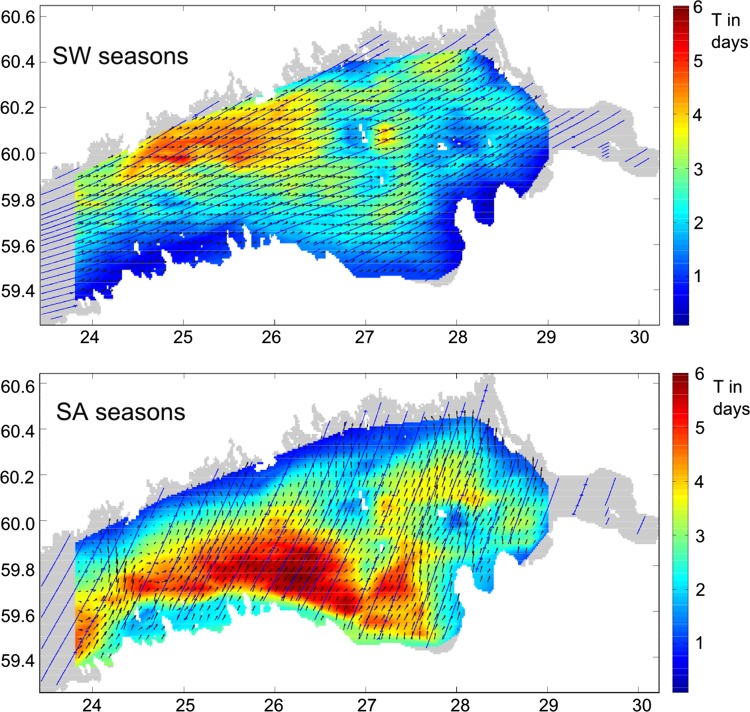
Spatial distribution of the average oil residence time at sea calculated using three-level nested NOAMOD and HBM models (Fig. 1) and the oil drift and fate model of DMI for the Gulf of Finland. *Solid lines* indicate streamlines of average 10 m winds *U*_10_, vectors reflect drift currents (sum of ocean currents and wind-induced oil drift calculated as 0.035*U*_10_) for summer and winter months (SW) and transitional seasons (spring–autumn, SA) in 1992–1994 (Murawski and Woge Nielsen 2013). ©Springer International Publishing. Reprinted with kind permission of Springer Science + Business Media

The existence and location of areas of reduced risk in terms of coastal pollution is established using statistical analysis of Lagrangian trajectories of single pollution parcels. The key outputs are the distributions of the probability for the parcels released in different sea areas to reach the nearshore regions and the time it takes (particle age or residence time at sea). The “climatological” maps of these quantities (Figs. 3, 4) mirror to some extent the distance from the coast but exhibit also obvious anisotropic features and demonstrate that a clearly larger environmental pressure is exerted on the eastern Baltic Sea coast (Fig. 3). They have usually low gradients in offshore areas and much higher gradients starting from a certain distance from the coast (Soomere et al. 2011c).

In several occasions such “climatological” distributions are almost meaningless. For the south-western Baltic Sea, two radically different regimes for the current-driven propagation of adverse impacts were identified: one for the typical inflow and another for outflow conditions (Lu et al. 2012). The maps reflecting the drift and fate of oil spills in the Gulf of Finland indicate the presence of two seasonal patterns of drift (Fig. 4). Somewhat counter-intuitively, one of these is valid for seasons hosting completely different forcing patterns—the windy winter season and calm summer season. The other one characterizes spring and autumn (Murawski and Woge Nielsen 2013).

The dependence of these maps on the model resolution was studied using the OAAS model with horizontal resolutions of 0.5, 1, and 2 nautical miles and with otherwise identical setup, forcing, and boundary conditions (Andrejev et al. 2011). The values of the mean probability of coastal hits and particle age for single realizations, their temporal behavior and cumulative values are highly correlated (*r* ≈ 0.98) for all resolutions. The standard deviations for the pointwise values of their distributions are almost the same for different resolutions. Also, the overall appearance of these maps, the location of the isolines and the areas of low probability and high particle age largely coincide for all resolutions.

A natural solution for a potentially dangerous activity, such as a drilling rig or a ship in distress, is a minimum of the relevant probability map or a maximum on the map of particle age or residence time of oil at sea. Such decisions have a relatively low uncertainty when these maps contain steep gradients in the area of interest (e.g., in the central narrow part of the Gulf of Finland where the optimum is sharp and clearly defined) whereas there is much freedom in domains where these gradients are small (Soomere et al. 2011c).

**Fig. 5:**
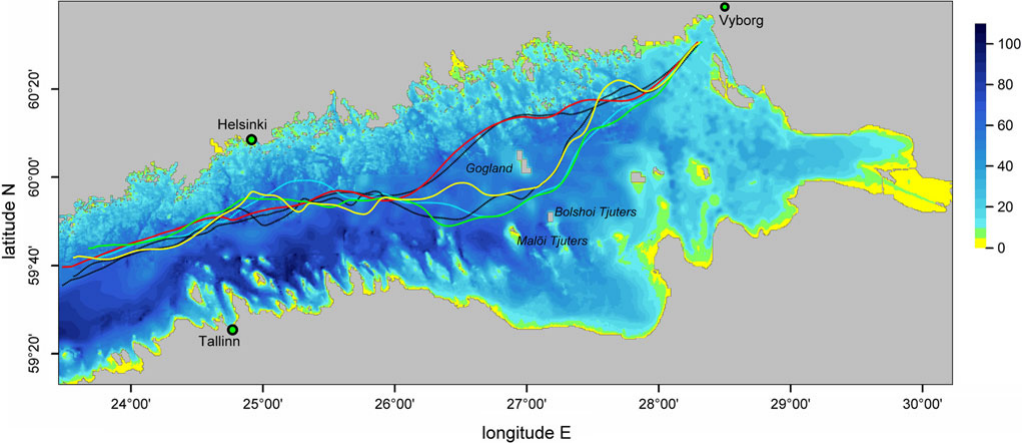
Optimum fairways from the Baltic Proper to Vyborg according to the spatial distributions of the probability for coastal hits (*solid lines*) and of the particle age (*dashed lines*) calculated using the OAAS model and “on-line” trajectory code of Andrejev et al. (2010) at resolutions of 2 (*red* and *black*), 1 (*green* and *cyan*), and 0.5 miles (*yellow* and *white*). The depth scale to the right of the map is given in meters (Andrejev et al. 2011)

There is a variety of different approaches to define the optimum fairway from such maps. The “fair way” dividing the risks equally between the opposite coasts is a natural (albeit local) and in many cases politically correct solution (Soomere et al. 2010; Lehmann et al. 2014). An elementary solution for elongated sea areas is to roughly follow the minima for the probabilities or the maxima for the particle age (Soomere et al. 2011c; Viikmäe et al. 2011). A similar result is achieved using a variation of the method of the smallest descent (Andrejev et al. 2010, 2011) that relies on the sequence of local decisions (Fig. 5). Although based on very simple applications, the resulting fairways may provide substantial environmental benefit. For example, in the Gulf of Finland their use may lead to a decrease by about 40 % in the probability of coastal pollution or almost double the typical residence time of the pollution at sea (Soomere et al. 2011b). The gain is smaller in the western Baltic Sea (10–30 % in terms of the probability of coastal hits within 10 days or an increase by about 1–2 days of the time it takes for the hit to occur, Lu et al. 2012) and open Baltic Sea (Höglund and Meier 2012; Lehmann et al. 2014).

The resulting fairways are almost insensitive with respect to the choice of the underlying measure of risk (Fig. 5). They are relatively sensitive with respect to the resolution of the circulation model, mainly because of a different representation of the coastline and islands at different resolutions (Andrejev et al. 2011). The sensitivity of the optimum fairway with respect to uncertainties in the underlying distributions can be implicitly estimated from the width of the area in which the relevant measure varies to some extent compared with its maximum or minimum. The width of such corridors is determined by the gradients of these measures and may vary substantially (more than ten times for sailing lines along the Gulf of Finland, Soomere et al. 2011c).

**Fig. 6:**
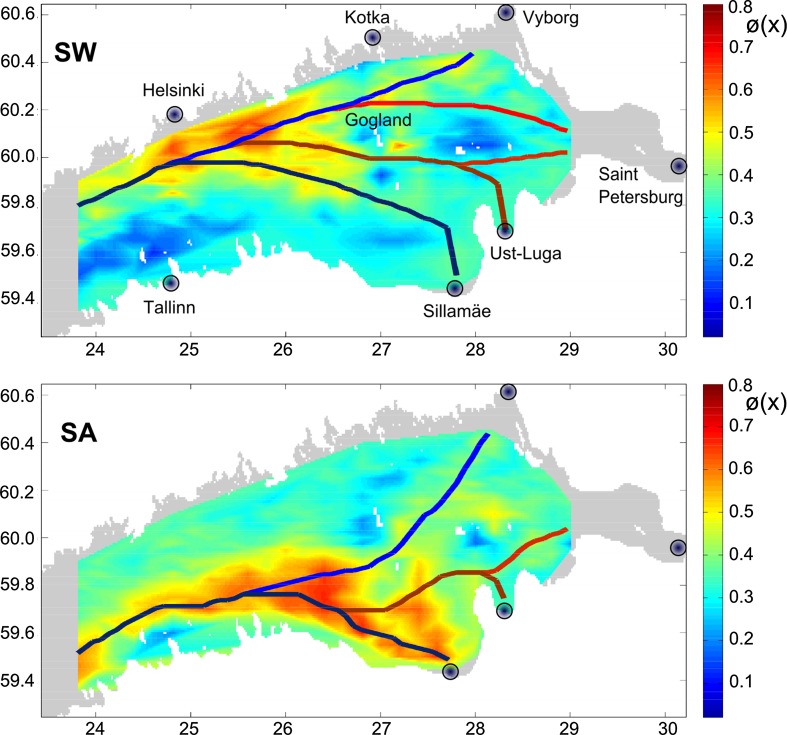
Optimum sailing lines in summer and winter (SW) and in spring–autumn (SA) seasons to Vyborg, Saint Petersburg, Ust-Luga, and Sillamäe constructed based on the aggregate cost function in which the residence time of oil at sea and the probability of coastal hit (both calculated using three-level nested NOAMOD and HBM models (Fig. 1) and the oil drift and fate model of DMI and presented in Fig. 4) have weights 2/3 and 1/3, respectively (Murawski and Woge Nielsen 2013). ©Springer International Publishing. Reprinted with kind permission of Springer Science + Business Media

The optimum sailing lines described above contain several meanders and are generally not suitable for ship traffic as course changes are typically fuel-consuming. Their excessive length is an additional source of environmental pressure through increased travel time and exhaust emissions. Murawski and Woge Nielsen (2013) incorporated into the fairway design the aggregate cost function accounting for both the residence time of oil at sea and the probability of oil landing (Fig. 4). They used a variation of an iterative Monte Carlo technique to alternatively optimize the path integral of the resulting cost function along possible sailing lines and the length of these lines. The resulting optimum, albeit relative, provides an acceptable option from the shipping industry viewpoint. Given the extensive seasonal variation of the counterparts of the cost function (Fig. 4), it is natural that the seasonally optimized sailing lines visit completely different sea areas (Fig. 6). The optimum fairways in transitional seasons (spring and autumn, called the SA design) are located in the southern or central part of the gulf whereas for summer- and wintertime (called the SW design) they are in the immediate vicinity of the Finnish archipelago. The SA design performs a little better than the SW design in terms of the normalized path integral of the cost function (Murawski and Woge Nielsen 2013). The somewhat longer SA route has much lower levels of environmental risks. Its performance is, however, rather low outside the spring and autumn seasons. The use of SW design in spring and autumn or vice versa leads to a considerable increase (by about 20–30 %) in the above-discussed aggregate measure. A combined design (not shown) performs reasonably well for all seasons. The choice between the proposed designs is ultimately the task of decision-makers as our estimates include an arbitrarily defined value of the vulnerable areas.

## Concluding Remarks

A new method has been developed to determine how various maritime activities can be made environmentally safer. The technique relies on the quantification of the marine space in terms of the potential of different sea areas to serve as a starting point of pollution that later impacts some vulnerable regions via current- and wind-driven transport. By placing maritime activities in the safest areas, the consequences of potential accidents can be minimized before they occur. This is an important element of science-based maritime spatial planning of the Baltic Sea toward sustainable use of the ecosystem and mitigation of the impact of controversial human activities.

Along with the development of algorithms for the identification of environmentally safer fairways, one of the challenges is the use of vast amounts of modeled information for solving dynamical problems in marine design. The use of Lagrangian trajectories as a sort of test elements of motion has made it possible to identify a number of features of transport of various adverse impacts by ocean currents which can be inferred neither from the analysis of classical (Eulerian) velocities nor from even massive measurements. An equally important development is the mapping of long-term behavior and dispersion properties of surface and subsurface currents in the Baltic Sea with the use of autonomous drifters.

The use of the optimized ship routes has a potential to substantially decrease the probability of coastal pollution or almost double the typical time it takes for the pollution to reach the coast. These estimates are, however, based on purely modeled features of ocean dynamics and Lagrangian transport of various adverse impacts in the surface layer and should be interpreted with some care. Also, the entire approach is based on certain statistical features of current- and wind-induced transport and the results therefore have a probabilistic nature. As the involved models, albeit they represent the state-of-the-art of the Baltic Sea modeling, have still many shortages, the derived estimates should be considered as preliminary ones. In particular, the inability of common models to replicate the statistics of velocity fields vividly calls for major improvements of the Baltic Sea circulation models.

It is thus likely that implementation of higher resolution models and improvements in model physics and quality of forcing data would lead to certain corrections of the presented estimates. First of all, more detailed resolving of eddy dynamics and at least a part of sub-mesoscale effects have a potential for considerable improvements. The demonstrated insensitivity of many results with respect to the underlying measures, model resolution, and on the particular spreading mechanism suggests that already the existing results have a clear value for environmental management of the Baltic Sea and at least qualitatively mirror the essence of pollution transport in this basin.

Although the results obtained so far only serve as a starting point of the relevant knowledge and competence, we still believe that the derived information is of vital importance for institutions responsible for environmental protection and maritime spatial planning. It is directly usable in the decision-making process in a crisis situation, e.g., about different search-and-rescue issues. The underlying studies have used several different models and have focused on various sub-basins of the Baltic Sea. Although the obtained estimates for environmentally safer fairways qualitatively match each other, future research should focus on a comprehensive intercomparison of the results. Such kind of information is needed for an assessment of the practical relevance of results and for the definition of future research together with more detailed knowledge about short-term variability of the underlying measures and associated ship routes. The subsequent challenges are linking the derived knowledge with policy toward the creation of the necessary societal, economical, legal, and political framework for the real implementation of the presented results.

## Glossary

**Fairway**: here the pathway of a ship on sea; also a navigable part of a water body.

**The Eulerian framework**, mostly used in circulation modelling, is a way of treating the properties of ocean currents for each fixed location in space as time passes.

**The Lagrangian specification** is a way of looking at fluid motion where the observer follows an individual water particle as it moves through space and time.

**Subgrid(-scale) motions** or processes are not resolved directly in numerical simulations due to the finite resolution of spatial grid and usually are parameterized using certain assumptions.

**Mesoscale motions** in the Baltic Sea have typical sizes from a few km to a few tens of km.
